# Cancer treatment delays among cancer patients living with HIV during the COVID‐19 pandemic in the United States

**DOI:** 10.1002/cam4.6489

**Published:** 2023-09-15

**Authors:** Ashley Khouri, Jessica Y. Islam, Nathan W. Van Bibber, Anna E. Coghill, Gita Suneja

**Affiliations:** ^1^ University of Utah School of Medicine Salt Lake City Utah USA; ^2^ Huntsman Cancer Institute Salt Lake City Utah USA; ^3^ Cancer Epidemiology Program H. Lee Moffitt Cancer Center & Research Institute Tampa Florida USA; ^4^ Department of Radiation Oncology University of Utah Salt Lake City Utah USA

**Keywords:** cancer, HIV and cancer, COVID‐19

## Abstract

**Background:**

The COVID‐19 pandemic led to care disruptions across the cancer continuum. It is unknown if immunosuppressed patients with cancer, who may be at higher risk for complications of SARS‐CoV‐2 infection, are disproportionately impacted. Thus, we aimed to compare delays in cancer treatment initiation between people living with HIV (PLWH) and cancer, the general cancer population (GCP), and patients with cancer and a history of solid organ transplant (SOT). Comparisons were made across the period 2 years preceding the pandemic versus the first year of the pandemic.

**Methods:**

We used data from a real‐world electronic health record‐derived de‐identified database (2018–2021) comprised of US patients with cancer from 800 sites of care across the country. We included patients with 19 different cancer types. We calculated time to cancer treatment initiation (TTI) as the difference between the date of cancer diagnosis and the earliest date that cancer treatment was recorded.

**Results:**

The sample included 181 PLWH, 65,073 GCP patients, and 195 patients with a SOT. Difference‐in‐difference regression models adjusted for age, sex, and presence of metastatic disease at cancer diagnosis revealed a significant increase in delayed TTI among PLWH compared to the GCP during COVID‐19 versus prior to COVID‐19, with delays increasing by approximately 1 month during the pandemic (DID: 32.6 days [8.9–56.3]; *p* = 0.007). The increase in TTI for PLWH was observed across treatment modalities, including surgery (DID: 55.1 [28.8–81.3], *p* < 0.001) and systemic therapy (DID: 30.4 [4.6–56.3], *p* = 0.021).

**Conclusions/Relevance:**

PLWH experienced significant delays in cancer treatment initiation after diagnosis during the first year of COVID‐19, delays that may negatively impact cancer outcomes. These data warrant patient and provider attention as the pandemic continues to impact the US healthcare system.

## Abstract 2

We aimed to determine whether already immunosuppressed populations, specifically people living with HIV and cancer and people living with a history of solid organ transplant and cancer, had more significant delays in cancer treatment initiation than the general cancer population during the COVID‐19 pandemic. To determine this, we used data from a real‐world electronic health record‐derived database with information from 800 sites of care around the United States.

## 1 BACKGROUND

With the emergence and spread of the novel coronavirus pandemic (COVID‐19), the provision of cancer care was adversely impacted in the United States. Utilization of cancer prevention services decreased significantly due to a variety of factors, including office closures during lockdown, patient fear of contracting COVID‐19 in the healthcare setting, or lack of transportation available to certain patients following widespread public transit closures.^1^, ^2^ In April of 2020, routine screenings for breast and colorectal cancer had dropped by 89.2% and 84.5%, respectively, compared to April of 2019.^3^ Many health systems suspended routine cancer screening and diagnostic procedures due to low personal protective equipment inventory or lack of established safety protocols.^3^ This interruption in care also extended to cancer treatment; within the first month of the pandemic, the U.S. Food and Drug Administration announced that 26 oncology medications were in short supply.^4^ Furthermore, cancer surgeries were impacted by staff shortages and lack of operating room space or hospital beds, and COVID‐related changes in guideline recommendations.^5^, ^6^

HIV care during COVID‐19 was also adversely impacted, with decreases in HIV testing and an increase in loss to follow‐up during HIV therapy.^7^, ^8^ Similar to patients with cancer, people living with HIV (PLWH) may have avoided healthcare interaction due to concern for contracting the virus as a consequence of immunosuppression.^9^ Outpatient HIV clinic office hours were reduced; for example, in South Carolina, 82% of HIV clinics were partially interrupted or completely closed during the beginning of the COVID‐19 outbreak.^8^ Furthermore, HIV service interruptions in that study were greatest in areas with higher percentages of uninsured patients, exacerbating a treatment access disparity that existed before COVID‐19.^8^

Despite published data documenting the impacts of COVID‐19 on cancer and HIV care separately, little is known about how COVID‐19 impacted cancer treatment among PLWH. Cancer treatment and outcomes disparities in PLWH are well‐documented in the pre‐pandemic era,^10^, ^11^, ^12^, ^13^, ^14^ so further interruptions to care in this vulnerable population during COVID‐19 and its continued impact on the US healthcare system could have had serious implications for clinical outcomes. In this study, we compared time to cancer treatment initiation (TTI) between cancer patients with no known underlying immunodeficiencies, and cancer patients with underlying HIV infection, both during the 2 years prior to COVID‐19 and during the first year of COVID‐19. We utilized, an electronic health record‐derived database from 800 sites of cancer care across the United States and hypothesized that delays in initiation of cancer treatment would be exacerbated for PLWH and cancer during the pandemic.

## 2 METHODS

### 2.1 Data source

We used de‐identified electronic health record (EHR)‐derived data from the Flatiron Health Database, which is comprised of patients from approximately 280 cancer clinics (800 sites of cancer care) across the US. This longitudinal database includes both structured and unstructured patient‐level, de‐identified data obtained from technology‐enabled chart abstraction.^15^, ^16^ We included data from the following analytic cohorts in this study: Advanced Melanoma, Diffuse Large B‐Cell Lymphoma, Early Breast Cancer, Advanced Endometrial Cancer, Follicular Lymphoma, Advanced Gastric Cancer, Hepatocellular Carcinoma, Advanced Renal Cell Carcinoma, Ovarian Cancer, Metastatic Pancreatic Adenocarcinoma, Metastatic Prostate Cancer, Small Cell Lung Cancer, Advanced Non‐Small Cell Lung Cancer, Metastatic Breast Cancer, Acute Myeloid Leukemia, Chronic Lymphocytic Leukemia, Metastatic Colorectal Cancer, Multiple Myeloma, and Advanced Head and Neck Cancer. For patients with multiple cancer diagnoses, the first cancer diagnosis was used.

### 2.2 Study groups

**FIGURE 1:**
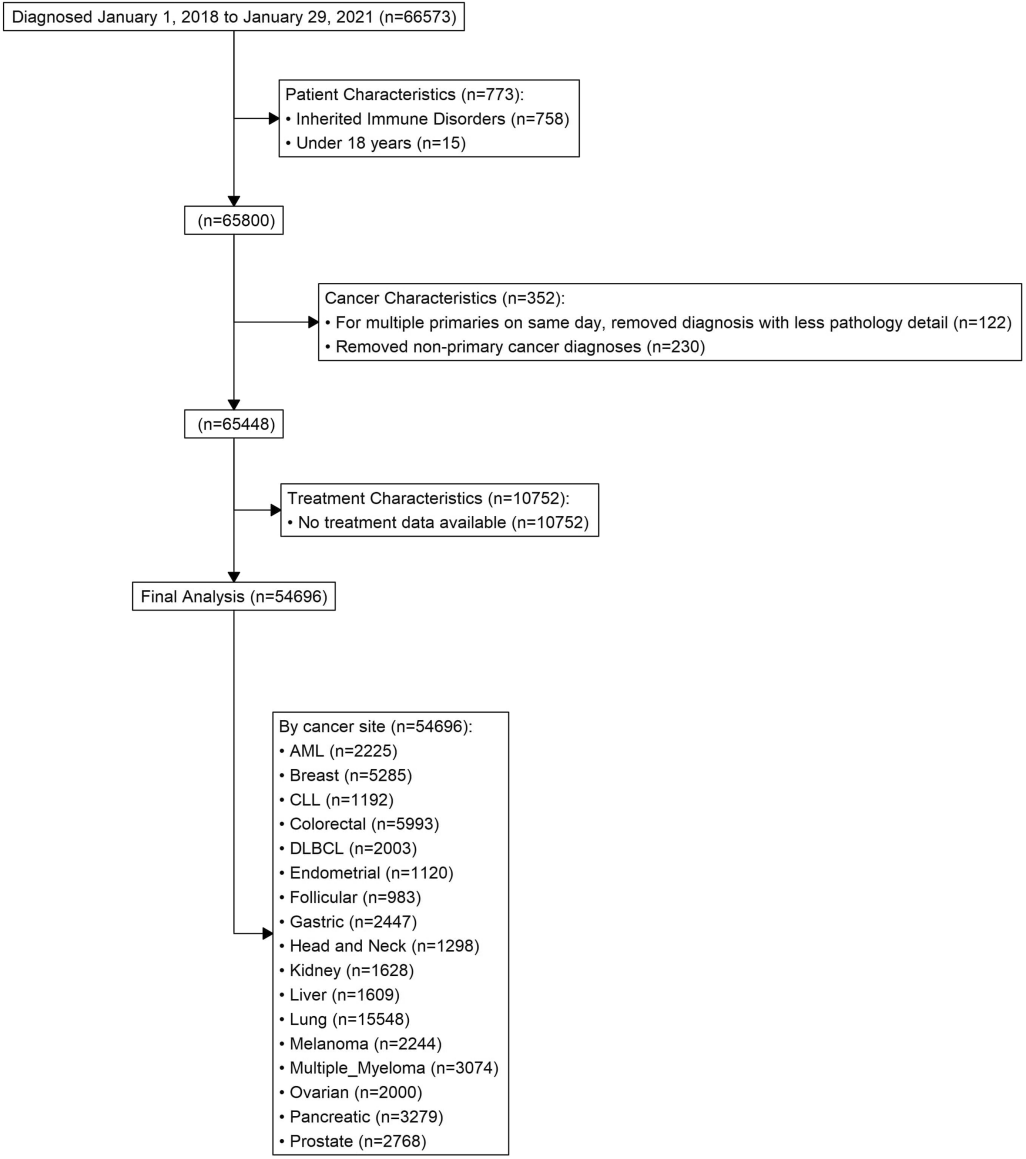
Patient Inclusion and Exclusion Criteria.

Three study groups were included in this analysis: people living with HIV (PLWH) and cancer, the general cancer population (GCP), defined as cancer patients without any underlying immunodeficiency, and cancer patients with a history of solid organ transplantation (SOT). We chose SOT patients as an additional, non‐HIV comparison group because these patients are administered immunosuppressive medications to prevent transplant rejection, allowing us to determine if any observed treatment delays were specific to PLWH or were generally present in immunosuppressed patients with cancer. The following International Classification of Diseases (ICD) codes were used to select PLWH: 42, 42.1, 42.2, 42.9, 43, 43.1, 43.2, 43.3, 43.9, 44, 44.9, 79.53, 795.8, B20, B97.35, O98.72, O98.71, O98.7. The following ICD codes were used to select patients with SOT: D89.81, Z94, Z94.0, Z94.1, Z94.2, Z94.3, Z94.4, Z94.5, Z94.6, Z94.7, Z94.8, Z94.82, Z94.83, Z94.89, Z94.9. Patients with hematopoietic transplantation were excluded from the SOT group. Patients with inherited immune disorders were excluded from the study. Our inclusion and exclusion criteria are displayed in Figure 1. HIV status was extracted from ICD‐10 codes with an associated date, which may or may not reflect HIV diagnosis date.

### 2.3 Primary outcome

The primary endpoint was time to treatment initiation (TTI). TTI was calculated as the difference in days between the date of the first cancer diagnosis in the real‐world database and the earliest date listed for administration of any of the following cancer treatments: (cell‐based treatment [CAR‐T therapy, Provenge], radiation therapy, surgery, systemic therapy [chemotherapy, immunotherapy, androgen deprivation therapy], transplant, other localized treatment [cryotherapy, transarterial chemoembolization]). We allowed a 14‐day window prior to diagnosis per oncologist‐defined, rule based Line of Therapy, such that if a patient was listed as both diagnosed and treated within 2 weeks in the EHR, the treatment lag was set to zero. All time to treatment analyses reported were conducted among patients with available treatment data; 16.4%, 19.9%, and 13.3% of the GCP, PLWH, and SOT groups, respectively, did not have treatment information in the database, either due to no treatment administration or to lack of treatment documentation.

### 2.4 Statistical analyses

The TTI was compared between study groups prior to COVID‐19 and during COVID‐19. The time period prior to COVID‐19 was defined as a diagnosis date between January 1, 2018 and March 12, 2020. The time period during COVID‐19 was defined as a diagnosis date between March 13, 2020, the day COVID‐19 was declared a national emergency in the United States, and January 29, 2021. We used *t*‐tests to compare median TTI between (1) the GCP and PLWH and cancer prior to COVID‐19, (2) the GCP and SOT recipients prior to COVID‐19, (3) the GCP and PLWH and cancer during COVID‐19, and (4) the GCP and SOT recipients during COVID‐19. A difference‐in‐differences analysis, which is a linear regression model that uses ordinary least squares to compare a change in the difference of an outcome between study groups over time, was performed to understand if differences in TTI between PLWH and the GCP changed across the pre‐ and during COVID‐19 time periods. This adjusted analysis included patient age, sex, and extent of disease (presence versus absence of metastatic disease at cancer diagnosis) in the regression model. Based on sample size, we were able to conduct DID analyses both overall and specific to two treatment types (surgery and systemic therapy). All data management was conducted using R,^17^ and statistical analyses were conducted using Stata V17. Flatiron Health datasets are de‐identified using the Expert Determination method outlined in HIPAA section 164.514(b).^15^, ^16^ Accordingly, this study was granted exemption by the Moffitt Cancer Center Scientific Review Board.

## 3 RESULTS

Sociodemographic and clinical characteristics of patients with cancer are outlined in Table 1. The sample included 65,073 cancer patients in the GCP group, 181 PLWH, and 195 with a history of SOT. In the GCP group, the majority were male (51.5%), White (62.1%), over 65 years of age at the time of cancer diagnosis (62.7%), and the most common cancers were lung cancer (30.9%), cancers of the gastrointestinal (GI) tract (28.8%), and lymphomas (11.1%). These same patterns were seen in the SOT group. Among PLWH, although the most common cancer types were similar, the racial and age distribution differed, with 50.3% of PLWH and cancer identifying as either Black (35.4) or Hispanic (14.9%), and the majority being males (72.4%) aged 45–64 years at cancer diagnosis (63.0%).

**TABLE 1:** Sociodemographic and clinical characteristics of the general cancer population, patients living with HIV and cancer, and patients with cancer and a history of solid organ transplant, Flatiron Health (2018–2021).

	General cancer population (*n* = 65,073)		Patients living with HIV and cancer (*n* = 181)		Patients with cancer with a history of solid organ transplant (*n* = 195)	
	No.	Col %	No.	Col %	No.	Col %
Age at diagnosis						
< 30 years	239	0.4	2	1.1	2	1.0
30–44	2366	3.6	12	6.6	5	2.6
45–64	21,661	33.3	114	63.0	89	45.6
65+	40,807	62.7	53	29.3	99	50.8
Sex						
Male	33,500	51.5	131	72.4	127	65.1
Female	31,569	48.5	50	27.6	68	34.9
Missing	4					
Race						
White	40,407	62.1	70	38.7	128	65.6
Black	5879	9.0	64	35.4	20	10.3
Asian	1539	2.4	0	0.0	10	5.1
Other	8736	13.4	33	18.2	25	12.8
Missing	8512	13.1	14	7.7	12	6.2
Ethnicity						
Hispanic or Latino	3509	5.4	27	14.9	15	7.7
Missing	61,564	94.6	154	85.1	180	92.3
Census region						
Northeast	10,203	17.7	19	15.2	11	16.2
Midwest	7553	13.1	8	6.4	14	20.6
South	28,940	50.3	85	68.0	33	48.5
West	10,793	18.8	13	10.4	10	14.7
Missing	7584		56		127	
History of smoking						
No	6154	9.5	12	6.6	16	8.2
Yes	24,662	37.9	74	40.9	40	20.5
Unknown / Missing	34,257	52.6	95	52.5	139	71.3
Body mass index						
Underweight	2091	3.2	11	6.1	4	2.1
Normal	16,640	25.7	60	33.3	55	28.4
Overweight	15,483	23.9	45	25.0	45	23.2
Obese	30,628	47.2	64	35.6	90	46.4
Missing	231		1		1	
ECOG performance score						
0	14,126	21.7	38	21.0	17	8.7
1	13,540	20.8	28	15.5	24	12.3
2	4793	7.4	13	7.2	7	3.6
3	1389	2.1	5	2.8	2	1.0
4	112	0.2	0	0.0	0	0.0
Missing	31,113	47.8	97	53.6	145	74.4
Solid tumor						
No	9177	14.1	31	17.1	37	19.0
Yes	55,896	85.9	150	82.9	158	81.0
Cancer type						
Lymphomas	7205	11.1	31	17.1	32	16.4
Myeloma	3342	5.1	6	3.3	5	2.6
Breast	5592	8.6	6	3.3	8	4.1
Gyn cancers	3362	5.2	7	3.9	6	3.1
GI cancers	18,717	28.8	52	28.7	101	51.8
Lung cancers	20,121	30.9	60	33.1	28	14.4
Prostate	2824	4.3	9	5.0	2	1.0
Melanoma	2401	3.7	1	0.6	5	2.6
Head & neck	1509	2.3	9	5.0	8	4.1
Year of diagnosis						
2018	25,276	38.8	66	36.5	89	45.6
2019	22,840	35.1	79	43.6	67	34.4
2020	16,523	25.4	34	18.8	39	20.0
2021 (January–February)	434	0.7	2	1.1	0	0.0
Metastatic cancer						
No	44,386	68.2	141	77.9	155	79.5
Yes	20,687	31.8	40	22.1	40	20.5
First cancer treatment type						
Cell‐based Tx	4	0.0	0	0.0	0	0.0
Other local Tx	633	1.2	6	4.1	29	17.2
Radiation	497	0.9	1	0.7	2	1.2
Surgery	7093	13.0	11	7.6	12	7.1
Systemic	46,140	84.8	127	87.6	100	59.2
Transplant	16	0.0	0	0.0	26	15.4
Missing	10,690		36		26	

**FIGURE 2:**
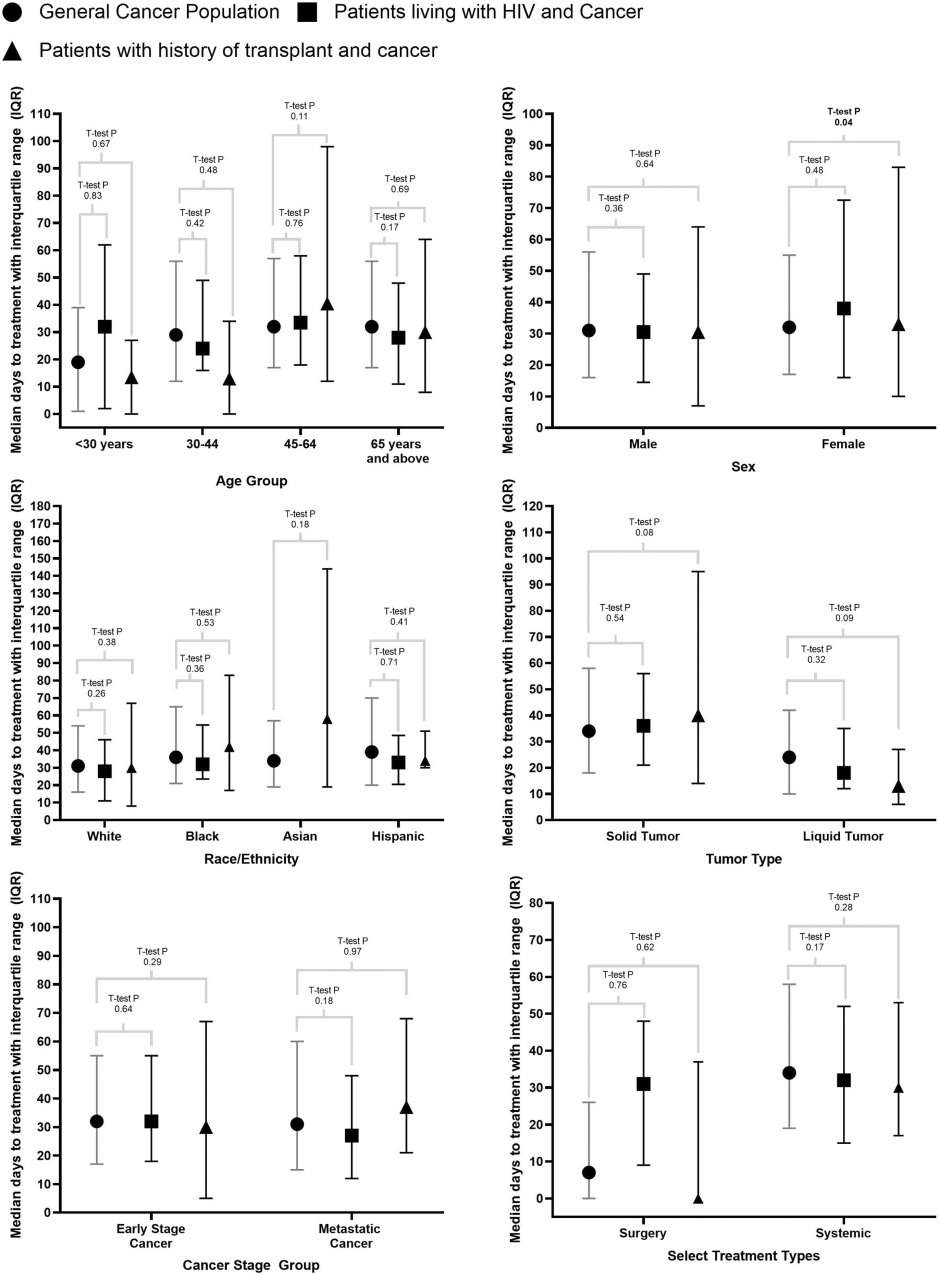
Time to cancer treatment initiation prior to COVID‐19 (January 2018 – March 2020). Differences between study groups were evaluated using a *t*‐test.

Figure 2 summarizes TTI by demographics in all three study groups in the 2 years prior to COVID‐19 in this set of cancer clinics. Overall, no statistically significant differences were observed in TTI between the GCP (median TTI 27.0 days) and PLWH (median TTI 27.0 days, *p* = 0.27), nor between the GCP and the SOT group (median 32.0 days, *p* = 0.39). A lack of significant differences in median TTI between PLWH and the GCP was consistent across most demographic and clinical groups.

**FIGURE 3:**
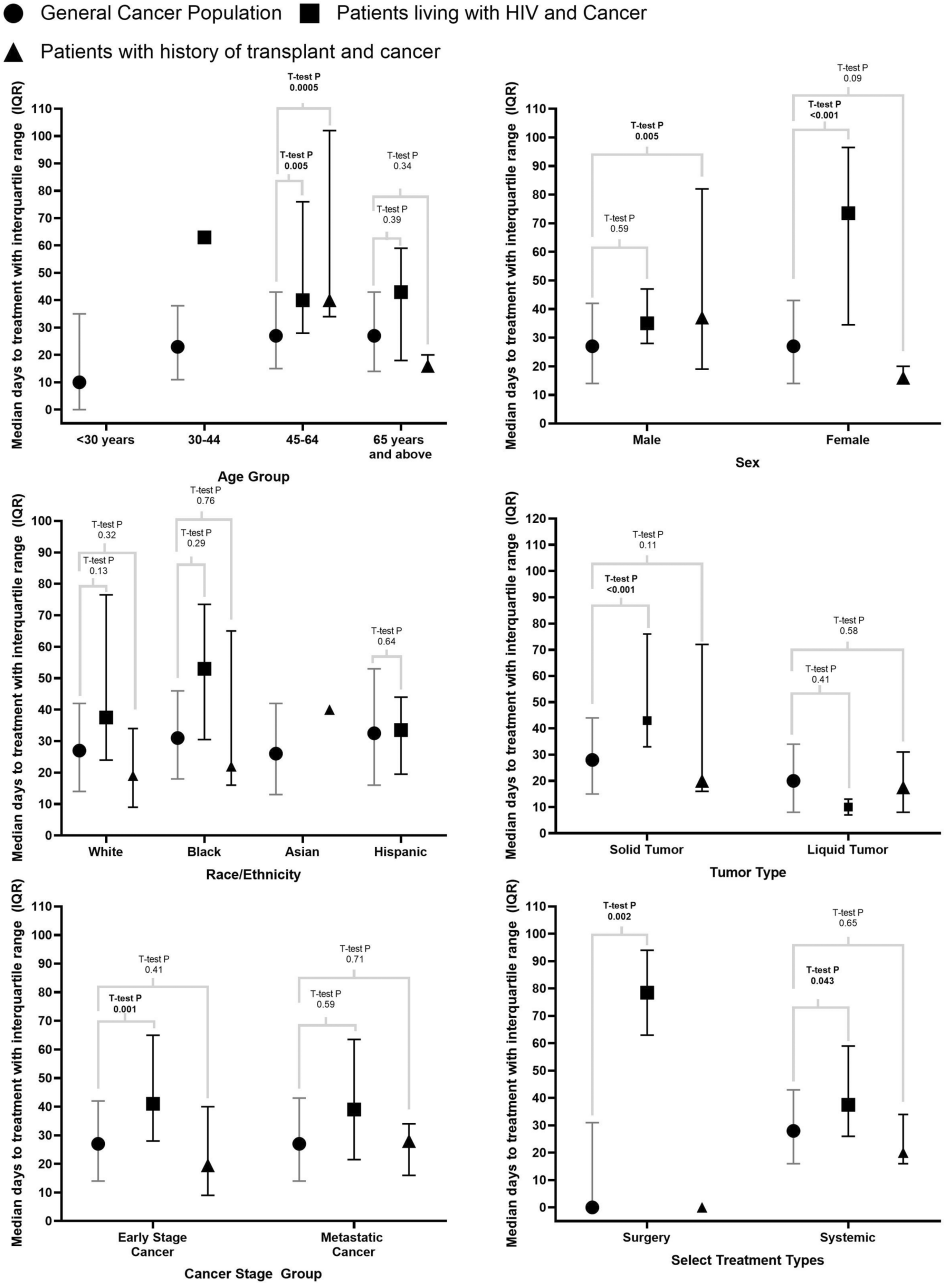
Time to cancer treatment initiation during COVID‐19 (March 2020–December 2020).

Figure 3 summarizes the TTI across study groups during the first year of the COVID‐19 pandemic. Overall, PLWH experienced statistically significant cancer treatment delays compared to the GCP (median TTI 41 days vs. 27 days, *p* = 0.0017). Females with HIV experienced pronounced cancer treatment delays, with a median TTI of 73.5 days, compared to 27.0 days for females in the GCP (*p* < 0.001). Statistically significant delays were also observed for smokers and obese PLWH, with median TTIs of 59.0 days and 65.0 days, respectively, compared to 30.0 and 31.0 days for smokers (p = 0.002) and obese patients from the GCP (*p* = 0.002). No difference was observed between the GCP and SOT recipients overall (median TTI 27 days vs. 20 days, *p* = 0.376). However, statistically significant cancer treatment delays were observed for SOT males compared to male GCP patients (median TTI 37.0 vs. 27.0, *p* = 0.005). In addition, both PLWH (median TTI 40 days) and SOT recipients (median TTI 40 days) ages 45–64 were more likely to experience cancer treatment delays compared to the GCP population aged 45–64 years (median TTI 27 days, *p* = 0.005 and 0.0005, respectively). This delay in treatment was not observed for patients age 65 and above.

Table 2 summarizes adjusted difference in difference regression estimates for changes in TTI between study groups prior to versus during COVID‐19. After adjusting for age, sex, and metastatic disease, when compared to the GCP, we observed a statistically significant increase in TTI delays among PLWH and cancer during the COVID‐19 pandemic. Specifically, the HIV‐associated TTI difference changed from a non‐significant −11.3 days prior to COVID‐19 to a significant +21.0 days (DID: 32.6, CI: 8.9–56.3; *p* = 0.007) during COVID‐19, approximately a 1‐month increase in TTI between PLWH and the GCP. The increase in the treatment delay between PLWH and the GCP during COVID‐19 was consistent when stratifying by patients who underwent surgery (DID: 55.1, 95% CI: 28.8–81.3, *p* < 0.001) and patients who received a systemic cancer therapy as their first treatment (DID: 30.4; 95% CI: 4.6–56.3, *p* = 0.021). We did not observe similar changes in the relative TTI between the SOT group and the GCP during COVID‐19 (Table 3). Furthermore, due to the difference in number of subjects per cohort, a sensitivity analysis was conducted based on matched samples in the GCP. Cohorts were matched on age at diagnosis, sex, and year of diagnosis. The results of the sensitivity analysis revealed the same findings as the primary analysis, and are shown in Supplementary Tables S1–S4.

**TABLE 2:** Difference‐in‐difference analysis for time to cancer treatment initiation prior to and during COVID‐19 by HIV status and treatment type.

	Prior to COVID‐19				During COVID‐19				Unadjusted DID	*p*‐value	Adjusted DID^a^	*p*‐value
	HIV	GCP	Difference	*p*‐value	HIV	GCP	Difference	*p*‐value				
Overall (*n* = 54,528)	51.7	63	−11.3	0.217	53.9	32.9	21	0.345	32.3 (CI: 8.7–55.9)	0.007	32.6 (CI: 8.9–56.3)	0.007
Type of treatment												
Surgery (*n* = 7116)	29.6	24.9	4.59	0.748	78.5	18.4	60.1	0.048	55.5 (28.6–82.3)	<0.001	55.1 (CI: 28.8–81.3)	<0.001
Systemic (*n* = 46,266)	53	68.6	−15.6	0.128	48.7	34.3	14.4	0.569	30.0 (CI: 3.9–56.2)	0.025	30.4 (CI: 4.6–56.3)	0.021

^a^ Adjusted for sex, age category, and metastatic disease (yes/no).

**TABLE 3:** Difference‐in‐difference analysis for time to cancer treatment initiation prior to and during COVID‐19 by SOT status and treatment type.

	Prior to COVID‐19				During COVID‐19				Unadjusted DID	*p*‐value	Adjusted DID^a^	*p*‐value
	SOT	GCP	Difference	*p*‐value	SOT	GCP	Difference	*p*‐value				
Overall (*n* = 54,696)	70.7	62.9	7.7	0.35	39	33	6.2	0.79	−1.49 (CI:‐30.85–27.85)	0.92	−1.42 (CI:‐30.69–27.8)	0.92
Type of treatment												
Systemic (*n* = 46,266)	54.6	68.6	−13.9	0.24	31	35	11.8	0.24	10.60 (CI:‐15.50–36.72)	0.43	11.23 (CI:‐15.01–37.48)	0.4

^a^ Adjusted for sex, age category, and metastatic disease (yes/no).

## 4 DISCUSSION

This study demonstrated that during the COVID‐19 pandemic, PLWH and cancer experienced significant delays in cancer treatment initiation compared to the general cancer population. We did not observe similar delays among cancer patients with a history of SOT, suggesting that the delay was specific to PLWH and not related to immunosuppression alone. This delay was observed for patients receiving both surgery and systemic therapy. These data warrant patient and provider attention, particularly as the pandemic continues to cause disruptions in healthcare access and cancer treatment utilization.

Delays in cancer treatment initiation have substantial clinical relevance. Several studies have shown that delays in surgical treatment of lung cancer, one of the most common tumors in our study population, are associated with decreased overall survival and increased mortality rates.^18^, ^19^, ^20^ For example, Ponholzer et al. reported that timely surgical resection in cT1‐staged lung cancer patients had a 76.3% risk reduction of death 5 years after surgery when compared to those receiving surgery greater than 60 days after diagnosis.^21^ Delays in receipt of surgery reached an average 79 days during COVID‐19 for PLWH in our analyses. Similar trends have been observed for delays in initiating systemic therapies. Delays in systemic chemotherapy after abreast or colon cancer diagnosis, for example, are associated with inferior overall survival.^22^, ^23^, ^24^ Therefore, although we did not analyze outcomes in this study due to limited available follow‐up time post‐pandemic, the observed treatment delays among PLWH during COVID‐19 are likely to precipitate adverse clinical outcomes in the coming years.

Cancer treatment delays during COVID‐19 should also be considered in the context of previously established cancer treatment and outcome disparities for PLWH. PLWH are more likely to be diagnosed with cancer at more advanced stages.^25^ PLWH and cancer are also less likely to receive cancer treatment, and are more likely to experience locoregional recurrence and elevated cancer‐specific mortality.^10^, ^11^, ^12^, ^13^, ^14^, ^25^, ^26^ More recently, data suggest that PLWH diagnosed with metastatic cancers, a population specifically recruited into the Flatiron database, are less likely to receive appropriate palliative care.^27^ Our data suggest that the already suboptimal provision of cancer care to PLWH has further deteriorated since the COVID‐19 pandemic and requires urgent prioritization to avoid poor cancer outcomes in this vulnerable patient population.

To our knowledge, this was the first study examining differences in time between cancer diagnosis and cancer treatment for PLWH during the COVID‐19 pandemic. We did not find a significant difference in TTI between PLWH and the GCP prior to COVID‐19. This lack of a treatment disparity differs from previously published literature,^10^, ^11^, ^12^, ^13^, ^14^ although prior studies primarily focused on receipt of any cancer treatment among PLWH rather than timely initiation of treatment. The large number of advanced and metastatic cancer patients included in the dataset (10 of the 19 cancer cohorts focused on advanced or metastatic disease) may have contributed to the similarity in TTI prior to COVID‐19. As such, the observed delay in cancer treatment during COVID‐19 among PLWH may have been even more extreme if we considered the full spectrum of cancer treatment centers across the U.S.

The delay in cancer treatment initiation observed for PLWH and cancer during COVID‐19 may reflect both patient and provider concerns about COVID‐19 symptomatology and severity in the setting of existing immune dysfunction. Recent studies show that while PLWH had similar COVID‐19 diagnosis rates as the general population, PLWH were more likely to be hospitalized and die from COVID‐19, particularly those with elevated viral load.^28^ Lower CD4+ counts are also associated with COVID‐19 ICU admissions, mechanical ventilation, and death.^28^ Mortality rates for PLWH with COVID‐19 were 9.4% overall and 51.5% among those admitted to an ICU.^29^ In summary, it is estimated that COVID‐19 provided an 80% excess risk of death among HIV‐infected individuals compared to those without diagnosed HIV/AIDS.^30^ While non‐cancer, non‐HIV medical co‐morbidities may account for some of this excess risk, rather than HIV itself, PLWH and oncologists may have still been hesitant to bring immunosuppressed patients into healthcare settings, particularly during the first year of the pandemic (2020) when knowledge surrounding COVID prevention, spread, and treatment was more limited.

A major strength of this study is the uniqueness of the Flatiron Health database. Flatiron Health is an EHR‐derived, real‐world clinical dataset with a substantial portion of cases with advanced disease, as well as documentation of multiple lines of cancer therapy. Flatiron Health de‐identified data originated from approximately 280 US cancer clinics (~800 sites of care). Limitations of the study include the overall low number of patients with HIV and history of SOT diagnosed with cancer in the examined 1‐year period after the emergence of COVID‐19. Additionally, heterogeneity of the cancer types included in the study population made granular comparisons challenging. Finally, 16.4%, 19.9%, and 13.3% of the GCP, PLWH, and SOT groups, respectively, did not have treatment information in the database and thus were excluded from analysis. This may have been due to no receipt of treatment or lack of data in the medical record due to receipt of treatment elsewhere. Nonetheless, the percentage of missing data was comparable among the three groups and so was unlikely to appreciably impact our key findings.

## 5 CONCLUSION

This is the first study to our knowledge that has examined delays in cancer treatment in PLWH during the COVID‐19 pandemic. We observed that during COVID‐19, delays in cancer treatment initiation for PLWH compared to the general cancer population were nearly 1 month greater. This delay was not observed among individuals with a history of SOT, suggesting a cancer treatment disparity specific to PLWH that warrants patient and provider attention.

## AUTHOR CONTRIBUTIONS

**Ashley Khouri:** Conceptualization (equal); data curation (equal); formal analysis (equal); investigation (equal); methodology (equal); project administration (equal); writing – original draft (lead); writing – review and editing (lead). **Jessica Y Islam:** Conceptualization (equal); data curation (equal); formal analysis (equal); methodology (equal); writing – review and editing (equal). **Nathan W. Van Bibber:** Data curation (equal); formal analysis (equal); methodology (lead). **Anna E. Coghill:** Conceptualization (equal); data curation (equal); formal analysis (equal); investigation (equal); methodology (equal); supervision (lead); writing – review and editing (equal). **Gita Suneja:** Conceptualization (equal); formal analysis (equal); investigation (equal); supervision (lead); writing – review and editing (equal).

## FUNDING INFORMATION

None.

## CONFLICT OF INTEREST STATEMENT

The authors declare no conflict of interest.

## Supporting information


Table S1–S4.

## Data Availability

The data that support the findings of this study are available on request from the corresponding author. The data are not publicly available due to privacy or ethical restrictions.
