# Supplementary material for: Cancer treatment delays among cancer patients living with HIV during the COVID‐19 pandemic in the United States
Source: Cancer Med. 2023 Sep 15;12(18):18717–28. doi: 10.1002/cam4.6489 (PMC10557907; doi:10.1002/cam4.6489)
Supplement: Supplementary file 1 — Table S1–S4. [file CAM4-12-18717-s001.docx]

Supplementary Table 1: Difference-in-difference analysis for time to treatment prior to and during COVID-19 by HIV status and treatment type among a 1:1 matched cohort by age, sex, and year of diagnosis (n=362)

|  | **Prior to COVID-19** | | | | **During COVID-19** | | | | **Unadjusted DID** | **P-Value** | **Adjusted DID*** | **P-Value** |
| --- | --- | --- | --- | --- | --- | --- | --- | --- | --- | --- | --- | --- |
|  | HIV | GOP | Difference | P-value | HIV | GOP | Difference | P-value |  |  |  |  |
| **Overall (n=301)** | 51.7 | 71.6 | -19.9 | 0.073 | 53.9 | 29 | 24.95 | 0.367 | 44.89  (CI: 12.85-76.92) | 0.006 | 44.52  (CI: 12.69-76.35) | 0.006 |
| **Type of Treatment** |  |  |  |  |  |  |  |  |  |  |  |  |
| **Surgery (n=33)** | 29.6 | 11.3 | 18.3 | 0.043 | 78.5 | 9 | 69.5 | <0.001 | 51.19  (CI: 17.45-84.94) | 0.004 | 49.97  (CI: 15.43-84.51) | 0.006 |
| **Systemic (n=255)** | 53 | 79.9 | -26.9 | 0.034 | 48.72 | 34.8 | 14.3 | 0.657 | 41.25  (CI: 4.68-77.83) | 0.027 | 40.66  (CI: 4.39-76.94) | 0.028 |

*Adjusted for age category, and metastatic disease (yes/no)

Supplementary Table 2: Difference-in-difference analysis for time to treatment prior to and during COVID-19 by SOT status and treatment type among a 1:1 matched cohort by age, sex, and year of diagnosis (n=362)

|  | | | | | | | | | | | | |  |
| --- | --- | --- | --- | --- | --- | --- | --- | --- | --- | --- | --- | --- | --- |
|  | **Prior to COVID-19** | | | | **During COVID-19** | | | | **Unadjusted DID** | **P-Value** | **Adjusted DID*** | | **P-Value** |
|  | SOT | GOP | Difference | P-value | SOT | GOP | Difference | P-value |  |  |  |  | |
| **Overall (n=333)** | 70.7 | 65.5 | 5.2 | 0.67 | 39.2 | 38.1 | 1.1 | 0.97 | -4.08  (CI: -39.97 - 31.81) | 0.82 | -1.81  (CI: -37.49- 33.88) | 0.92 | |
| **Type of Treatment** |  |  |  |  |  |  |  |  |  |  |  |  | |
| **Systemic (n=46,266)** | 54.6 | 74.3 | -19.6 | 0.18 | 31 | 36.4 | -5.4 | 0.86 | 14.22  (CI: -21.09-49.52) | 0.43 | 15.42  (CI: -20.97-51.81) | 0.41 | |

*Adjusted for age category, and metastatic disease (yes/no)

Supplementary Table 3: Sociodemographic and clinical characteristics of matched (age, sex, year of diagnosis) Cohort of GCP and SOT Cancer Patient Populations

|  | **General Cancer Population (n=195)** | | **Patients with Cancer with a History of Solid Organ Transplant (n=195)** | | **Total (n=390)** | |
| --- | --- | --- | --- | --- | --- | --- |
|  | No. | Col % | No. | Col % | No. | Col % |
| **Age at diagnosis** |  |  |  |  |  |  |
| <30 years | 2 | 1 | 2 | 1 | 4 | 1 |
| 30-44 | 5 | 2.6 | 5 | 2.6 | 10 | 2.6 |
| 45-64 | 89 | 45.6 | 89 | 45.6 | 178 | 45.6 |
| 65+ | 99 | 50.8 | 99 | 50.8 | 198 | 50.8 |
| **Sex** |  |  |  |  |  |  |
| Male | 127 | 65.1 | 127 | 65.1 | 254 | 65.1 |
| Female | 68 | 34.9 | 68 | 34.9 | 136 | 34.9 |
| **Race** |  |  |  |  |  |  |
| White | 126 | 64.6 | 128 | 65.6 | 254 | 65.1 |
| Black | 17 | 8.7 | 20 | 10.3 | 37 | 9.5 |
| Asian | 5 | 2.6 | 10 | 5.1 | 15 | 3.8 |
| Other | 24 | 12.3 | 25 | 12.8 | 49 | 12.6 |
| Missing | 23 | 11.8 | 12 | 6.2 | 35 | 9 |
| **Ethnicity** |  |  |  |  |  |  |
| Hispanic or Latino | 10 | 5.1 | 15 | 7.7 | 25 | 6.4 |
| Missing | 185 | 94.9 | 180 | 92.3 | 365 | 93.6 |
| **Census Region** |  |  |  |  |  |  |
| Northeast | 22 | 11.3 | 11 | 5.6 | 33 | 8.5 |
| Midwest | 27 | 13.8 | 14 | 7.2 | 41 | 10.5 |
| South | 91 | 46.7 | 33 | 16.9 | 124 | 31.8 |
| West | 23 | 11.8 | 10 | 5.1 | 33 | 8.5 |
| Missing | 32 | 16.4 | 127 | 65.1 | 159 | 40.8 |
| **History of Smoking** |  |  |  |  |  |  |
| No | 18 | 9.2 | 16 | 8.2 | 34 | 8.7 |
| Yes | 84 | 43.1 | 40 | 20.5 | 124 | 31.8 |
| Unknown / Missing | 93 | 47.7 | 139 | 71.3 | 232 | 59.5 |
| **Body Mass Index** |  |  |  |  |  |  |
| Underweight | 7 | 3.6 | 4 | 2.1 | 11 | 2.8 |
| Normal | 51 | 26.2 | 55 | 28.2 | 106 | 27.2 |
| Overweight | 43 | 22.1 | 45 | 23.1 | 88 | 22.6 |
| Obese | 94 | 48.2 | 90 | 46.2 | 184 | 47.2 |
| Missing | 0 | 0 | 1 | 0.5 | 1 | 0.3 |
| **ECOG Performance Score** |  |  |  |  |  |  |
| 0 | 44 | 22.6 | 17 | 8.7 | 61 | 15.6 |
| 1 | 35 | 17.9 | 24 | 12.3 | 59 | 15.1 |
| 2 | 13 | 6.7 | 7 | 3.6 | 20 | 5.1 |
| 3 | 1 | 0.5 | 2 | 1 | 3 | 0.8 |
| Missing | 102 | 52.3 | 145 | 74.4 | 247 | 63.3 |
| **Solid Tumor** |  |  |  |  |  |  |
| No | 31 | 15.9 | 37 | 19 | 68 | 17.4 |
| Yes | 164 | 84.1 | 158 | 81 | 322 | 82.6 |
| **Cancer Type** |  |  |  |  |  |  |
| Lymphomas | 20 | 10.3 | 32 | 16.4 | 52 | 13.3 |
| Myeloma | 14 | 7.2 | 5 | 2.6 | 19 | 4.9 |
| Breast | 13 | 6.7 | 8 | 4.1 | 21 | 5.4 |
| Gyn Cancers | 8 | 4.1 | 6 | 3.1 | 14 | 3.6 |
| GI cancers | 59 | 30.3 | 101 | 51.8 | 160 | 41 |
| Lung cancers | 63 | 32.3 | 28 | 14.4 | 91 | 23.3 |
| Prostate | 4 | 2.1 | 2 | 1 | 6 | 1.5 |
| Melanoma | 8 | 4.1 | 5 | 2.6 | 13 | 3.3 |
| Head and neck | 6 | 3.1 | 8 | 4.1 | 14 | 3.6 |
| **Year of Diagnosis** |  |  |  |  |  |  |
| 2018 | 89 | 45.6 | 89 | 45.6 | 178 | 45.6 |
| 2019 | 67 | 34.4 | 67 | 34.4 | 134 | 34.4 |
| 2020 | 39 | 20 | 39 | 20 | 78 | 20 |
| **Metastatic Cancer** |  |  |  |  |  |  |
| No | 142 | 72.8 | 155 | 79.5 | 297 | 76.2 |
| Yes | 53 | 27.2 | 40 | 20.5 | 93 | 23.8 |
| **First Cancer Treatment Type** |  |  |  |  |  |  |
| Other Local Tx | 5 | 2.6 | 29 | 14.9 | 34 | 8.7 |
| Radiation | 1 | 0.5 | 2 | 1 | 3 | 0.8 |
| Surgery | 23 | 11.8 | 12 | 6.2 | 35 | 9 |
| Systemic | 135 | 69.2 | 100 | 51.3 | 235 | 60.3 |
| Transplant | 0 | 0 | 26 | 13.3 | 26 | 6.7 |
| Missing | 31 | 15.9 | 26 | 13.3 | 57 | 14.6 |

Supplementary Table 4: Sociodemographic and clinical characteristics of matched (age, sex, year of diagnosis) Cohort of GCP and HIV Cancer Patient Populations

|  | **General Cancer Population**  **(n=181)** | | **Patients living with HIV and Cancer (n=181)** | | **Total (n=362)** | |
| --- | --- | --- | --- | --- | --- | --- |
|  | No. | Col % | No. | Col % | No. | Col % |
| **Age at diagnosis** |  |  |  |  |  |  |
| <30 years | 2 | 1.1 | 2 | 1.1 | 4 | 1.1 |
| 30-44 | 12 | 6.6 | 12 | 6.6 | 24 | 6.6 |
| 45-64 | 114 | 63 | 114 | 63 | 228 | 63 |
| 65+ | 53 | 29.3 | 53 | 29.3 | 106 | 29.3 |
| **Sex** |  |  |  |  |  |  |
| Male | 131 | 72.4 | 131 | 72.4 | 262 | 72.4 |
| Female | 50 | 27.6 | 50 | 27.6 | 100 | 27.6 |
| **Race** |  |  |  |  |  |  |
| White | 107 | 59.1 | 70 | 38.7 | 177 | 48.9 |
| Black | 15 | 8.3 | 64 | 35.4 | 79 | 21.8 |
| Asian | 10 | 5.5 | 0 | 0 | 10 | 2.8 |
| Other | 25 | 13.8 | 33 | 18.2 | 58 | 16 |
| Missing | 24 | 13.3 | 14 | 7.7 | 38 | 10.5 |
| **Ethnicity** |  |  |  |  |  |  |
| Hispanic or Latino | 10 | 5.5 | 27 | 14.9 | 37 | 10.2 |
| Missing | 171 | 94.5 | 154 | 85.1 | 325 | 89.8 |
| **Census Region** |  |  |  |  |  |  |
| Northeast | 24 | 13.3 | 19 | 10.5 | 43 | 11.9 |
| Midwest | 28 | 15.5 | 8 | 4.4 | 36 | 9.9 |
| South | 72 | 39.8 | 85 | 47 | 157 | 43.4 |
| West | 31 | 17.1 | 13 | 7.2 | 44 | 12.2 |
| Missing | 26 | 14.4 | 56 | 30.9 | 82 | 22.7 |
| **History of Smoking** |  |  |  |  |  |  |
| No | 20 | 11 | 12 | 6.6 | 32 | 8.8 |
| Yes | 70 | 38.7 | 74 | 40.9 | 144 | 39.8 |
| Unknown / Missing | 91 | 50.3 | 95 | 52.5 | 186 | 51.4 |
| **Body Mass Index** |  |  |  |  |  |  |
| Underweight | 4 | 2.2 | 11 | 6.1 | 15 | 4.1 |
| Normal | 54 | 29.8 | 60 | 33.1 | 114 | 31.5 |
| Overweight | 43 | 23.8 | 45 | 24.9 | 88 | 24.3 |
| Obese | 79 | 43.6 | 64 | 35.4 | 143 | 39.5 |
| Missing | 1 | 0.6 | 1 | 0.6 | 2 | 0.6 |
| **ECOG Performance Score** |  |  |  |  |  |  |
| 0 | 47 | 26 | 38 | 21 | 85 | 23.5 |
| 1 | 40 | 22.1 | 28 | 15.5 | 68 | 18.8 |
| 2 | 10 | 5.5 | 13 | 7.2 | 23 | 6.4 |
| 3 | 0 | 0 | 5 | 2.8 | 5 | 1.4 |
| Missing | 84 | 46.4 | 97 | 53.6 | 181 | 50 |
| **Solid Tumor** |  |  |  |  |  |  |
| No | 24 | 13.3 | 31 | 17.1 | 55 | 15.2 |
| Yes | 157 | 86.7 | 150 | 82.9 | 307 | 84.8 |
| **Cancer Type** |  |  |  |  |  |  |
| Lymphomas | 16 | 8.8 | 31 | 17.1 | 47 | 13 |
| Myeloma | 13 | 7.2 | 6 | 3.3 | 19 | 5.2 |
| Breast | 16 | 8.8 | 6 | 3.3 | 22 | 6.1 |
| Gyn Cancers | 4 | 2.2 | 7 | 3.9 | 11 | 3 |
| GI cancers | 68 | 37.6 | 52 | 28.7 | 120 | 33.1 |
| Lung cancers | 47 | 26 | 60 | 33.1 | 107 | 29.6 |
| Prostate | 0 | 0 | 9 | 5 | 9 | 2.5 |
| Melanoma | 12 | 6.6 | 1 | 0.6 | 13 | 3.6 |
| Head and neck | 5 | 2.8 | 9 | 5 | 14 | 3.9 |
| **Year of Diagnosis** |  |  |  |  |  |  |
| 2018 | 66 | 36.5 | 66 | 36.5 | 132 | 36.5 |
| 2019 | 79 | 43.6 | 79 | 43.6 | 158 | 43.6 |
| 2020 | 34 | 18.8 | 34 | 18.8 | 68 | 18.8 |
| 2021  (January-February) | 2 | 1.1 | 2 | 1.1 | 4 | 1.1 |
| **Metastatic Cancer** |  |  |  |  |  |  |
| No | 123 | 68 | 141 | 77.9 | 264 | 72.9 |
| Yes | 58 | 32 | 40 | 22.1 | 98 | 27.1 |
| **First Cancer Treatment Type** |  |  |  |  |  |  |
| Other Local Tx | 5 | 2.8 | 6 | 3.3 | 11 | 3 |
| Radiation | 1 | 0.6 | 1 | 0.6 | 2 | 0.6 |
| Surgery | 22 | 12.2 | 11 | 6.1 | 33 | 9.1 |
| Systemic | 128 | 70.7 | 127 | 70.2 | 255 | 70.4 |
| Missing | 25 | 13.8 | 36 | 19.9 | 61 | 16.9 |
